# Leflunomide/teriflunomide inhibit Epstein-Barr virus (EBV)-induced lymphoproliferative disease and lytic viral replication

**DOI:** 10.18632/oncotarget.17863

**Published:** 2017-05-15

**Authors:** Andrea Bilger, Julie Plowshay, Shidong Ma, Dhananjay Nawand ar, Elizabeth A. Barlow, James C. Romero-Masters, Jillian A. Bristol, Zhe Li, Ming-Han Tsai, Henri-Jacques Delecluse, Shannon C. Kenney

**Affiliations:** ^1^ Department of Oncology, School of Medicine and Public Health, University of Wisconsin-Madison, Madison, Wisconsin, USA; ^2^ Department of Medicine, School of Medicine and Public Health, University of Wisconsin-Madison, Madison, Wisconsin, USA; ^3^ Department Cellular and Molecular Biology, School of Medicine and Public Health, University of Wisconsin-Madison, Madison, USA; ^4^ Department of Cellular and Molecular Pathology, School of Medicine and Public Health, University of Wisconsin-Madison, Madison, Wisconsin, USA; ^5^ Sanofi Pharmaceuticals, Cambridge, Massachusetts, USA; ^6^ Rocky Mountain Infectious Disease Specialists, Aurora, Colorado, USA; ^7^ Department of Cancer Biology and Immunology, Dana-Farber Cancer Institute and Department of Medicine, Harvard Medical School, Cambridge, Massachusetts, USA; ^8^ Joint DKFZ Inserm Unit U1074, German Cancer Center (DKFZ), Heidelberg, Germany

**Keywords:** therapy, lymphoma, lymphoproliferative disease, humanized mouse model, FDA-approved

## Abstract

EBV infection causes mononucleosis and is associated with specific subsets of B cell lymphomas. Immunosuppressed patients such as organ transplant recipients are particularly susceptible to EBV-induced lymphoproliferative disease (LPD), which can be fatal. Leflunomide (a drug used to treat rheumatoid arthritis) and its active metabolite teriflunomide (used to treat multiple sclerosis) inhibit *de novo* pyrimidine synthesis by targeting the cellular dihydroorotate dehydrogenase, thereby decreasing T cell proliferation. Leflunomide also inhibits the replication of cytomegalovirus and BK virus via both “on target” and “off target” mechanisms and is increasingly used to treat these viruses in organ transplant recipients. However, whether leflunomide/teriflunomide block EBV replication or inhibit EBV-mediated B cell transformation is currently unknown. We show that teriflunomide inhibits cellular proliferation, and promotes apoptosis, in EBV-transformed B cells *in vitro* at a clinically relevant dose. In addition, teriflunomide prevents the development of EBV-induced lymphomas in both a humanized mouse model and a xenograft model. Furthermore, teriflunomide inhibits lytic EBV infection *in vitro* both by preventing the initial steps of lytic viral reactivation, and by blocking lytic viral DNA replication. Leflunomide/teriflunomide might therefore be clinically useful for preventing EBV-induced LPD in patients who have high EBV loads yet require continued immunosuppression.

## INTRODUCTION

Epstein-Barr virus (EBV) is a human herpesvirus that infects the majority of the world's population and causes infectious mononucleosis [1]. Like all herpesviruses, EBV infects cells in both latent and lytic forms. EBV-infected humans sustain life-long latent viral infection within the memory B cell compartment, and periodically shed infectious viral particles into the saliva [2]. Once recovered from their initial infection, immunocompetent hosts only rarely develop symptomatic illness related to EBV. Nevertheless, EBV efficiently transforms primary B cells *in vitro*, and is associated with a variety of different types of B-cell lymphomas and EBV-induced lymphoproliferative disease (EBV-LPD) in humans, particularly in immunocompromised hosts [3].

Latent EBV infection is sufficient to transform B cells *in vitro* in the absence of any lytic viral gene expression [4], and the major EBV transforming proteins (EBNA2 and LMP1) are expressed during latent infection [3]. Nevertheless, both uncontrolled latent and lytic infection likely contribute to the development of EBV-LPD in immunosuppressed patients. Patients who require pharmacologic immunosuppression, such as bone marrow and solid organ transplant patients, have a high risk of developing EBV-LPD, particularly when they have high EBV loads in the blood [5]. High EBV loads in immunosuppressed patients are usually caused by a greatly increased number of latently-infected B cells; in some cases an increased number of lytically-infected cells also contribute to high viral load [6]. Drugs that can either prevent the proliferation of latently-infected B cells, and/or the production of infectious EBV particles, may help to prevent the development of EBV-LPD in immunosuppressed patients with high EBV loads. Valacyclovir, which inhibits viral replication when metabolized to acyclovir, has been shown to reduce the number of EBV-infected cells in healthy volunteers [7]. However, it remains controversial whether drugs that specifically inhibit lytic (but not latent) EBV infection effectively prevent and/or treat EBV-LPD in immunosuppressed patients [8–11].

Leflunomide, an immunosuppressive drug approved for the treatment of rheumatoid arthritis since 1998, is increasingly also used to treat human cytomegalovirus (HCMV) and BK virus infection in transplant patients [12–14]. Teriflunomide, the active metabolite of leflunomide, is approved for treatment of multiple sclerosis [15]. The “on target” effect of leflunomide and teriflunomide, which occurs at low doses, is mediated through inhibition of the cellular dihydroorotate dehydrogenase (DHODH) enzyme [16]. DHODH is required for *de novo* pyrimidine synthesis (but not for pyrimidine synthesis mediated by the salvage pathway), and “on target” effects of the leflunomide/teriflunomideare reversed *in vitro* by supplementing the media with uridine, which restores *de novo* pyrimidine synthesis. Lymphocytes are particularly dependent upon *de novo* pyrimidine synthesis for their proliferation [17], and the major “on target” immunosuppressive effect of leflunomide/teriflunomide is thought to be due to decreased T cell proliferation. In addition to decreasing the amount of pyrimidine-based nucleotides available for DNA/RNA synthesis, drugs that inhibit DHODH activity globally decrease the level of O-linked GlcNAcylate-modified proteins through an “on-target” effect [18]. Diffuse large B-cell lymphoma (DLBCL) cell lines and primary DLBCL tumor cells have higher levels of nuclear O-GlcNAcylate-modified proteins than do normal B-cells, and the levels of these proteins correlate with DLBCL cell growth and survival [19].

Higher doses of leflunomide (still easily achieved in patients) have been proposed to have numerous additional “off-target” effects *in vitro* [12, 20–23]. Inhibition of HCMV lytic replication by leflunomide is likely mediated through an “off target” effect, since it is not reversed by uridine supplementation, although the exact mechanism(s) by which the drug acts on HCMV replication are not clear [22, 24, 25]. Higher dose leflunomide has also been shown to inhibit the proliferation and survival of chronic lymphocytic leukemia (CLL) cells *in vitro* through “off-target” effects on signaling pathways such as NF-kappa B and STAT3 [23].

However, whether leflunomide or teriflunomide can be used to inhibit lytic viral replication in EBV-infected B cells (similar to its effect on HCMV), or to prevent proliferation and/or survival of latently infected B cells (similar to its effect on CLL cells), is not known.Here we have investigated whether therapeutically relevant levels of teriflunomide inhibit the lytic form of EBV replication and/or block proliferation of latently-infected B cells *in vitro*. In addition, we have used two different mouse models to ask if these drugs inhibit the growth of EBV-induced lymphomas *in vivo* at non-toxic doses. We show that teriflunomide not only blocks the lytic form of EBV infection (and hence could be used to prevent transmission of the virus from cell to cell), but also greatly decreases the growth of latently infected, EBV-induced lymphomas *in vivo*. These results suggest that leflunomide/teriflunomide may be useful for preventing (and potentially treating) EBV-induced LPD in immunosuppressed patients with high EBV loads.

## RESULTS

### Teriflunomide inhibits proliferation of EBV-transformed B cells *in vitro*

**Figure 1:**
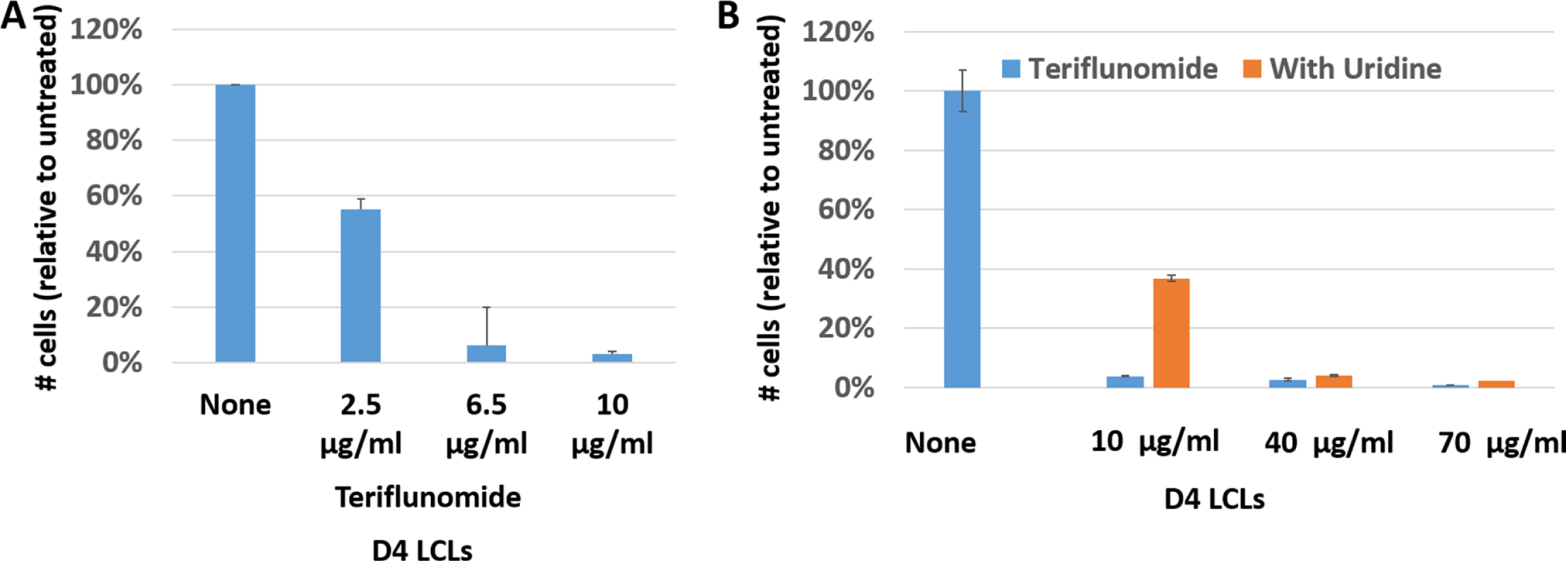
Teriflunomide inhibits proliferation of EBV-transformed B cells *in vitro* D4 LCLs were plated at 2 × 10^5^ cells/ml, treated with teriflunomide or DMSO vehicle control on day 0, and harvested on day 7. (**A**) Cells were counted with a hemocytometer using Trypan blue staining and counts were normalized to the DMSO control. (**B**) Cells were treated with DMSO control or with teriflunomide, given alone or one hour after treatment with 150 μM uridine. Relative cell titers were determined using CellTiter-Glo (Promega).

Leflunomide/teriflunomide inhibit the proliferation of T lymphocytes and can interfere with the replication of some viruses. We therefore examined whether the active leflunomide metabolite, teriflunomide (A771726), affects proliferation of EBV-transformed human B cells *in vitro*. As shown in Figure 1A and 1B, teriflunomide prevented proliferation of the EBV-transformed lymphoblastoid cell line (LCL), D4, *in vitro;* similar results were obtained with a second independently derived lymphoblastoid cell line, M81-Luc (data not shown). The addition of uridine to the media in cells treated with a very low dose of teriflunomide (10 μg/ml, which is well below the target plasma concentration of 40–80 μg/ml for treatment of rheumatoid arthritis patients) partially reversed this anti-proliferative effect (Figure 1B). At higher doses of drug (40 and 70 μg/ml) the anti-proliferative effect was not reversed by uridine. These results indicate that teriflunomide prevents proliferation of EBV-transformed human B cells through both “on target” and “off target” mechanisms. Importantly, since patients can tolerate a teriflunomide level as high as 100 μg/ml [26], high-dose leflunomide/teriflunomide could potentially be used to inhibit the proliferation of latently EBV-infected B cells in humans, thereby taking advantage of both the “on-target” and “off-target” effects of the drug.

### Teriflunomide alters EBV latency protein expression in EBV-transformed B cells *in vitro*

**Figure 2:**
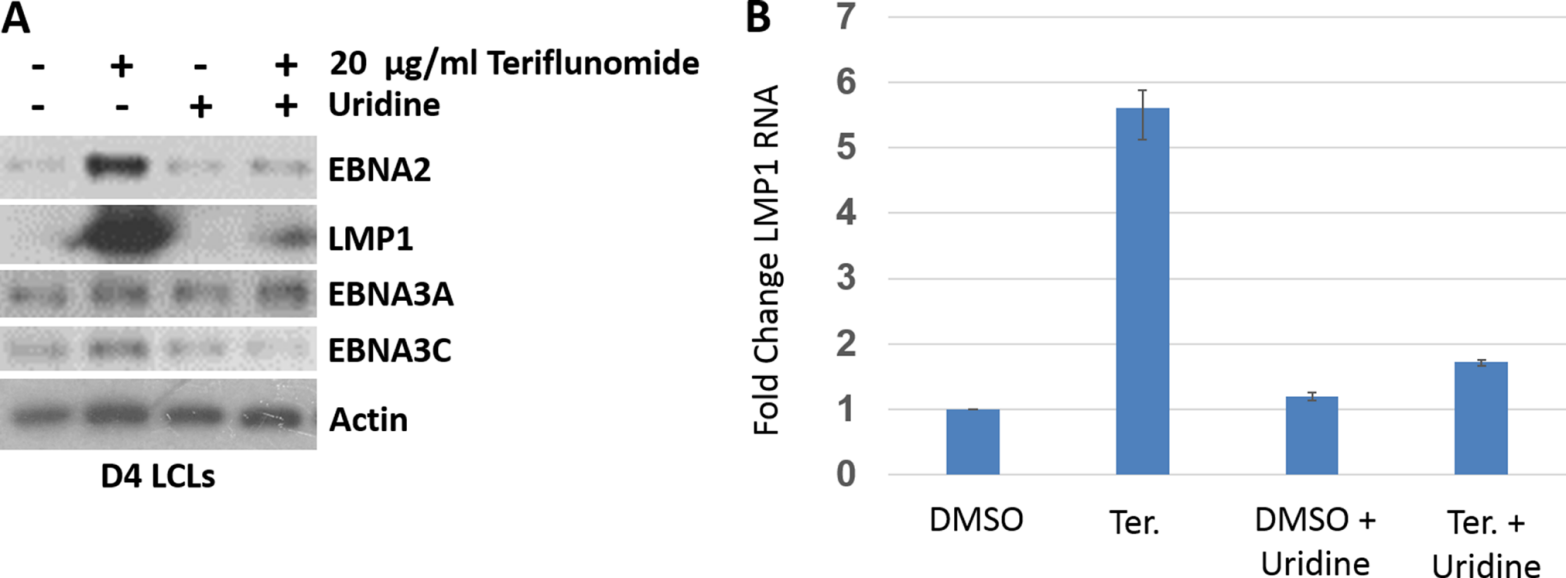
Teriflunomide alters EBV latency protein expression in EBV-transformed B cells *in vitro* (**A**) D4 LCLs were treated with teriflunomide or with DMSO control, each given alone or with 150 μM uridine. Cells were harvested after 7 days of treatment. Immunoblot analysis was performed using antibodies to the EBV latency proteins shown. β-actin was used as a loading control. (**B**) RNA was prepared from D4 LCLs treated with teriflunomide or DMSO control for 7 days, reverse-transcribed, and assessed using quantitative PCR with LMP1-specific primers. The level of LMP1 transcript in untreated cells is set as 1. The data represent one experiment done in duplicate.

To explore potential mechanisms(s) by which teriflunomide halts proliferation of EBV-transformed LCLs, we examined whether the drug alters expression of EBV latency proteins known to be required for LCL survival and proliferation *in vitro*. As shown in Figure 2A, teriflunomide treatment increased expression of two different EBV transforming proteins, EBNA2 (which mimics the effects of Notch signaling) [27] and LMP1 (which mimics the effects of CD40 signaling) [28], while not significantly affecting the expression of two other essential EBV transforming proteins (EBNA3A and EBNA3C). Furthermore, the ability of teriflunomide to increase EBNA2 and LMP1 expression was observed using a low dose (20 μg/ml) of drug and was reversed by uridine treatment, suggesting that it is due to an “on target” effect. The level of LMP1 transcript was also significantly higher in teriflunomide-treated versus untreated cells, and this effect was reversed by uridine treatment (Figure 2B). Interestingly, although low level LMP1 expression is required for proliferation and survival of EBV-transformed LCLs, higher level LMP1 expression (as little as twice that of normal level expression) inhibits B cell proliferation [29]. Taken together, these results suggest that excessive production of LMP1 may contribute to the anti-proliferative effect of teriflunomide in EBV-transformed LCLs.

### Teriflunomide induces p53 expression and apoptosis in LCLs *in vitro*

**Figure 3:**
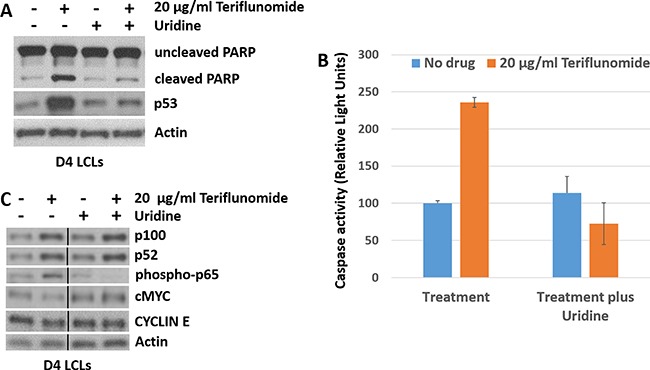
Teriflunomide induces p53 expression and apoptosis in LCLs *in vitro* D4 LCLs were treated with teriflunomide or with DMSO control, each given alone or with 150 μM uridine. Cells were harvested after 7 days of treatment (12 hours after the final drug treatment). (**A**) and (**C**) Immunoblot analysis was performed using antibodies to the cell survival and proliferation factors shown. β-actin was used as a loading control. (**B**) D4 LCLs were treated with teriflunomide or DMSO for 5 days. After one additional day of culture in the absence of drug, cells were assayed for caspase 3/7 activity using Caspase Glo (Promega).

We next determined whether teriflunomide's effect on LCL growth is associated with increased apoptosis. The tumor suppressor protein, p53, plays a critical role in promoting both cell cycle arrest and apoptosis, and teriflunomide has been shown to increase p53 levels by blocking pyrimidine biosynthesis [30]. As shown in Figure 3A, teriflunomide treatment of LCLs increased p53 expression. Furthermore, teriflunomide treatment increased the level of cleaved PARP (a marker for apoptosis) [31], and the amount of activated caspases 3 and 7 (Figure 3B), and these effects were reversed by uridine supplementation. Thus, teriflunomide induces apoptosis of EBV-transformed B cells through its on-target mechanism, and this effect is likely at least partially mediated by increased p53 expression.

### Teriflunomide does not inhibit canonical or non-canonical NF-kB signaling in EBV-transformed LCLs, and has little effect on c-Myc and cyclin E expression

Teriflunomide has been reported to induce killing of chronic lymphocytic leukemia (CLL) B cells *in vitro* through off-target effects including suppression of the canonical NF-kB cell survival pathway [23]. Since EBV-transformed LCLs require LMP1-induced NF-kB for their survival [28], we examined whether teriflunomide inhibits the canonical or non-canonical NF-kB pathways in LCLs. As shown in Figure 3C, teriflunomide treatment did not reduce the level of phosphorylated p65 (a marker of canonical NF-kB signaling), or the amount of p52 produced by cleavage of p100 (a marker of the non-canonical NF-kB pathway; reviewed in [32]). Indeed, the levels of these factors increased somewhat, likely reflecting the ability of increased LMP1 to induce both types of NF-kB pathways [28].

In addition, since EBNA2-induced c-Myc expression is required for LCL proliferation [33], we examined whether teriflunomide treatment reduces expression of either c-Myc, or its target gene, cyclin E [34], in LCLs. As shown in Figure 3C, there was no significant change in c-Myc or cyclin E levels in teriflunomide-treated cells. These results suggest that teriflunomide does not globally inhibit NF-kB survival pathways, or c-Myc-mediated transcription, in EBV-transformed B cells.

### Teriflunomide inhibits lytic EBV replication in B cells

**Figure 4:**
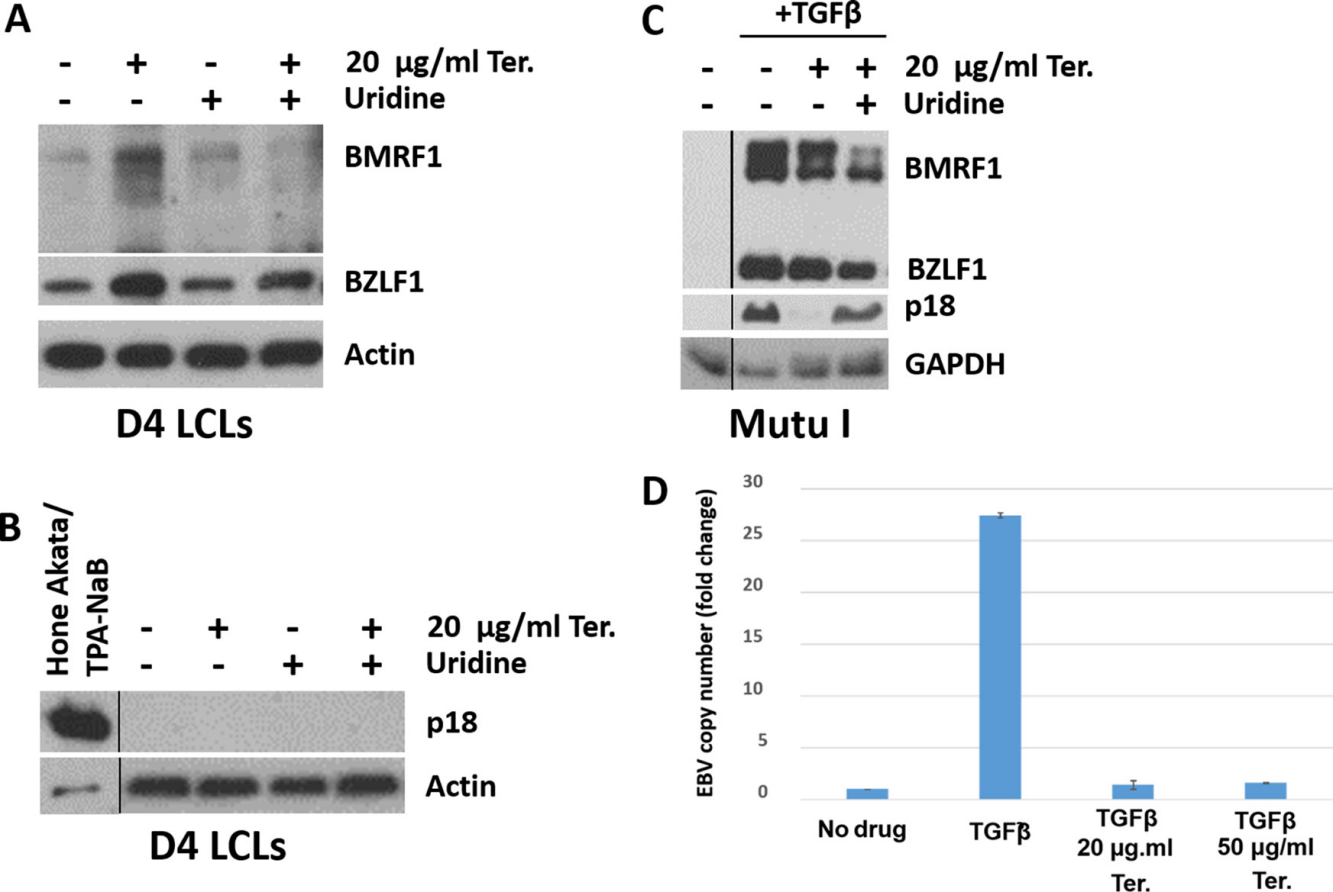
Teriflunomide inhibits lytic EBV replication in B cells Immunoblot analysis was performed to detect the immediate early lytic protein BZLF1, the early lytic protein BMRF1, or the late lytic protein p18 in control or teriflunomide-treated cells, with or without uridine supplementation. β-actin or GAPDH was used as a loading control. (**A**, **B**) D4 LCLs were treated with teriflunomide or with DMSO control, each given alone or with 150 μM uridine, for 7 days. The same extracts were used in panels A and B. Control HONE-Akata cells were treated for 3 days with 20 ng/ml TPA and 3 mM sodium butyrate, which are known to induce the late lytic EBV protein p18 in these cells. (**C**) Mutu I Burkitt cells were treated with teriflunomide or with DMSO control, given alone or with 150 uM uridine, for 3 days. TGFβ was given as indicated on day 0 to induce lytic activation. (**D**) DNA was extracted from untreated or TGFβ-treated Mutu I cells (treated with or without teriflunomide as indicated), and quantitative PCR was used to determine the amount of EBV DNA, normalized to the amount of β-globin DNA.

Given the ability of leflunomide to inhibit lytic HCMV replication [22, 24], we determined whether teriflunomide alters the amount of lytic EBV replication in EBV-infected B cell lines. Consistent with its ability to increase p53 expression, which has previously been shown to promote enhanced early lytic EBV protein expression [35, 36], teriflunomide increased levels of both the EBV immediate-early lytic protein, BZLF1, and the early lytic protein, BMRF1, in LCLs (Figure 4A). In spite of this activation of early lytic EBV proteins, the late viral capsid antigen, p18, was not expressed in teriflunomide-treated LCLs (Figure 4B). This result suggests that fully lytic EBV infection (required to transmit the virus from cell to cell) may be blocked by teriflunomide treatment.

We next examined the effect of teriflunomide treatment on lytic EBV protein expression and viral DNA replication in TGF beta-treated Burkitt lymphoma cells (Figure 4C). As previously described [36], TGF beta treatment of EBV-infected Burkitt cells activated expression of the EBV BZLF1 and BMRF1 immediate-early/early lytic proteins, as well as the EBV late lytic protein, p18. While low dose (20 μg/ml) teriflunomide treatment did not prevent TGF beta-mediated activation of early lytic EBV proteins, it blocked induction of the late EBV protein, p18 (Figure 4C), and this effect was largely reversed by uridine supplementation.

Since late EBV gene expression (but not early lytic gene expression) requires the lytic form of EBV DNA replication [37], we next asked whether teriflunomide decreases the amount of intracellular EBV DNA replication in TGFβ-treated Mutu I cells. As shown in Figure 4D, TGFβ treatment increased the amount of EBV DNA in Mutu I cells, and teriflunomide (even at the low dose, 20 μg/ml) prevented this. Together, these results reveal that teriflunomide inhibits the lytic form of EBV DNA replication in B cells at doses easily achieved in humans, and that this effect is at least partially mediated by its effect on *de novo* pyrimidine synthesis.

### Teriflunomide inhibits the earliest step of lytic EBV reactivation in response to B-cell receptor ligation, or phorbol ester treatment

**Figure 5:**
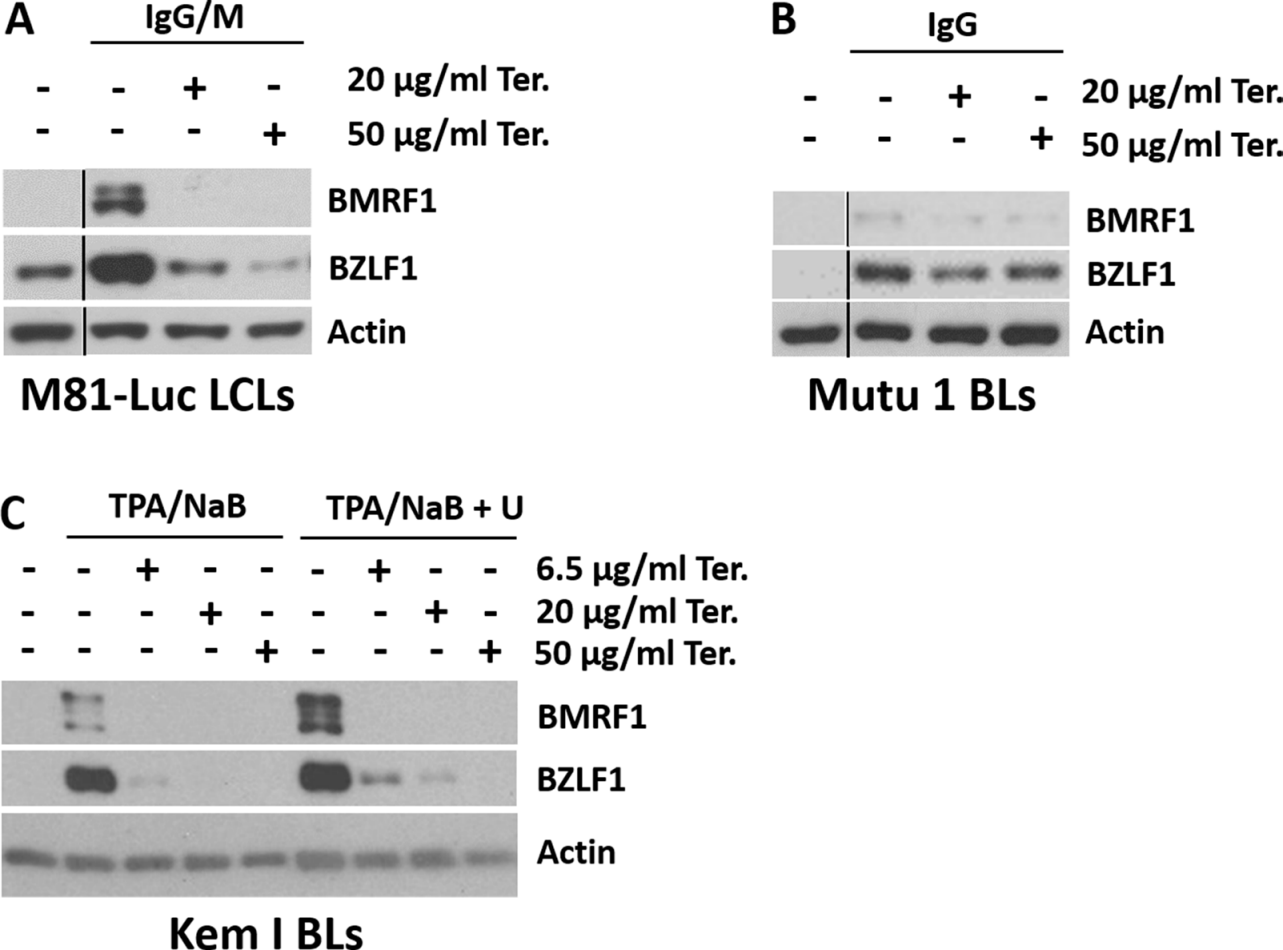
Teriflunomide inhibits the earliest step of lytic EBV reactivation in response to B-cell receptor ligation or phorbol ester Western analysis was performed to detect the immediate early lytic protein BZLF1 or the early lytic protein BMRF1 after lytic induction of control or teriflunomide-treated cells, with or without uridine supplementation. β-actin was used as a loading control. (**A**) M81-Luc LCLs were induced with a combination of 10 μg/ml anti-human IgG and 6.8 μg/ml anti-human IgM; (**B**) Mutu I Burkitt cells were induced with 10 μg/ml anti-human IgG; and (**C**) Kem I Burkitt cells were induced with a combination of 20 ng/ml TPA and 3 mM sodium butyrate.

In the course of the TGFβ treatment studies, we discovered that teriflunomide prevents the ability of some lytic-inducing stimuli to activate a much earlier stage of lytic EBV reactivation (i.e., immediate-early BZLF1 protein expression). In particular, teriflunomide reduced immediate-early lytic protein BZLF1 and early lytic protein BMRF1 expression in response to B-cell receptor (BCR) ligation in both an EBV-transformed LCL line (Figure 5A) and an EBV-infected Burkitt line (Figure 5B). BCR stimulation by antigen is thought to be a biologically relevant mechanism by which the virus undergoes lytic reactivation in humans (reviewed in [35]). Low-dose teriflunomide also dramatically blocked BZLF1 and BMRF1 lytic protein expression in EBV-infected Burkitt cells treated with the combination of a phorbol ester (TPA) and an HDAC inhibitor (sodium butyrate; Figure 5C). Since the BMRF1 protein acts as the EBV DNA polymerase processivity factor, and is required for the lytic form of EBV DNA replication [38–40], no lytic EBV replication can occur in the absence of BMRF1 expression. Interestingly, teriflunomide blockade of early lytic EBV protein expression in response to TPA/sodium butyrate treatment and BCR stimulation was not reversed by uridine supplementation (Figure 5C and data not shown), although it occurred at low doses of the drug. Together, these results indicate the low dose teriflunamide inhibits the ability of EBV to lytically replicate its genome by multiple different mechanisms, some of which involve on-target effects, and others which are mediated through off-target effects.

### Teriflunomide inhibits the growth of EBV-transformed LCLs in a xenograft mouse model

Given the ability of teriflunomide to inhibit proliferation of LCLs *in vitro*, we next asked whether teriflunomide treatment *in vivo* inhibits the growth of M81-Luc LCLs in a xenograft mouse model. For these experiments, we used an LCL transformed with an EBV virus (M81 strain) that contains a luciferase gene inserted into the viral genome under the control of the constitutively active HCMV IE gene promoter, such that optical scans could be used to measure tumor sizes at different time points. NSG mice were injected subcutaneously in each flank with 5 million M81-Luc LCL cells (mixed with matrigel), and then treated with teriflunomide (20 mg/ kg ip, three times a week), starting at day 8 after injection of the cells. The experiment was ended when untreated tumors reached the maximum allowable size for xenografts.

**Figure 6:**
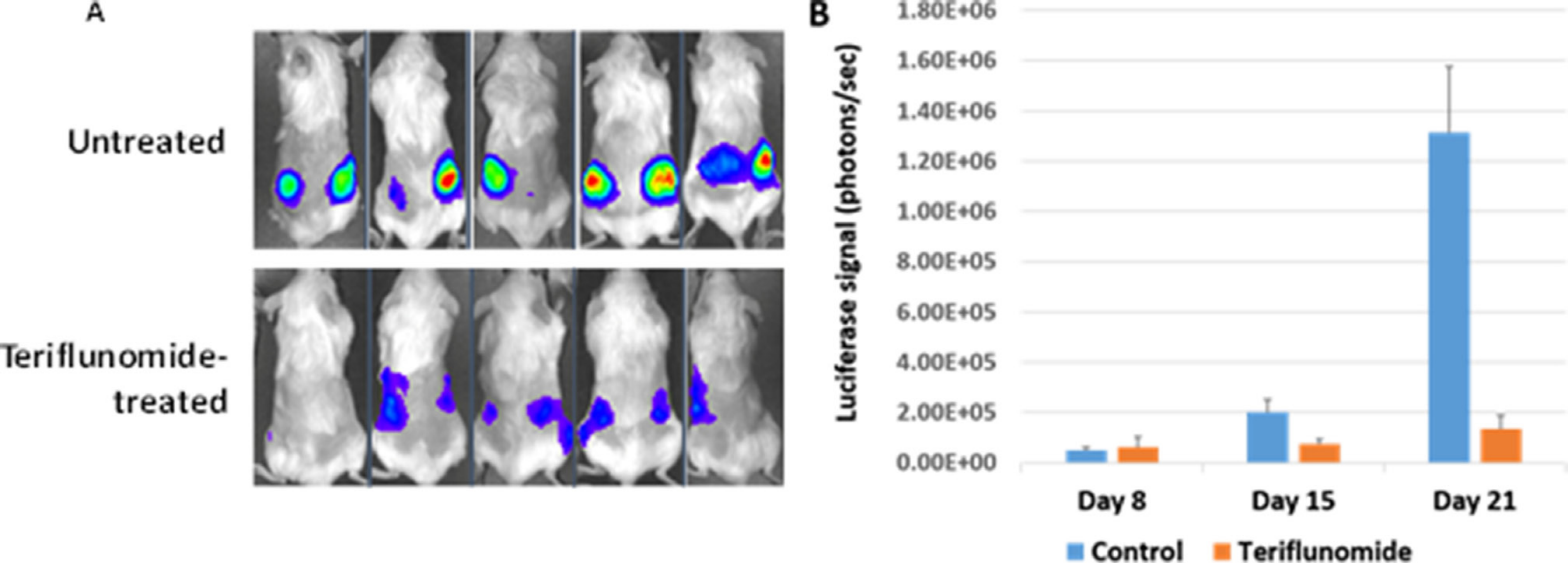
Teriflunomide inhibits the growth of EBV-transformed LCLs in a xenograft mouse model NSG mice were injected subcutaneously in the flanks with 5 × 10^6^ M81-Luc LCL cells on day 0, then treated with 20 mg/kg teriflunomide or PBS 3 times per week starting on day 8, and euthanized on day 21. (**A**) On days 8, 15, and 21, mice were given 150 mg/kg luciferin intraperitoneally and scanned using an IVIS imaging system. (**B**) The light produced during scanning was quantified using Living Image Software.

The amount of luciferase activity at each cell injection site was measured at several time points. As shown in Figure 6A and 6B, teriflunomide treatment dramatically reduced luciferase activity (a measurement of tumor size) relative to saline-treated animals, and did not cause any obvious toxicity in animals.

### Leflunomide prevents the development of EBV-induced lymphomas in a cord-blood humanized mouse model

To further explore the potential utility of leflunomide/teriflunomide treatment for preventing EBV-induced lymphoproliferative disease in immunocompromised patients, we examined its effect in a recently developed cord-blood humanized mouse model. In this model, CD34-depeleted cord blood cells are infected with EBV particles for 1 hour *in vitro*, and then cord blood cells are injected intraperitoneally into NSG mice. As previously described by our group [41], both EBV-infected B cells and human T cells engraft into the spleen and lymph nodes in this model. Human T cells initially act to inhibit the growth of EBV-induced B cell lymphomas in this system, even though most animals eventually die from such lymphomas [42]. If the ability of leflunomide/teriflunomide to prevent T cell proliferation is greater than its effect on EBV-induced lymphoma formation, then leflunomide could potentially promote lymphomas in this model via its on-target immunosuppressive effects on T cells.

**Figure 7:**
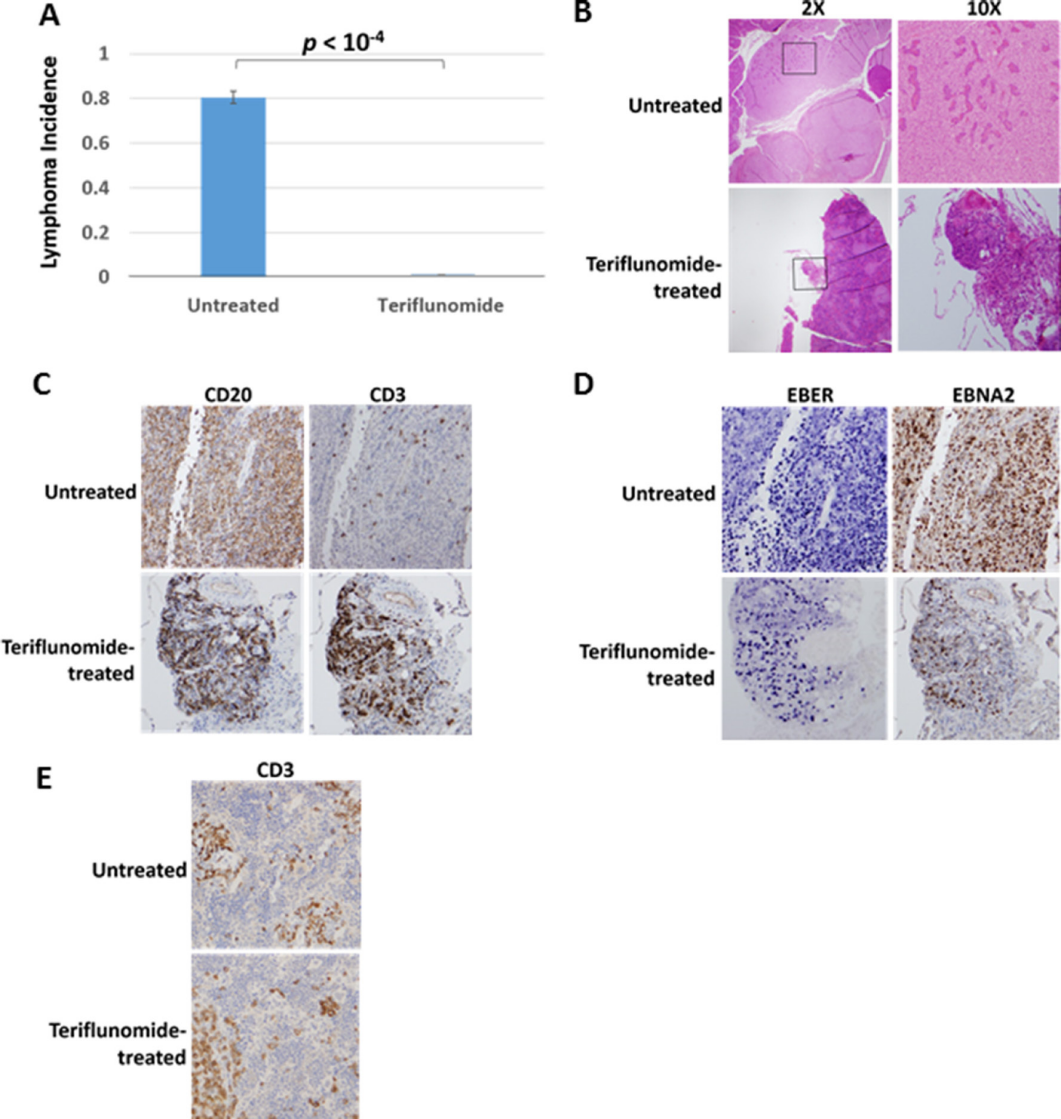
Teriflunomide prevents the development of EBV-induced lymphomas in a cord blood-humanized mouse model In two independent experiments, NSG mice were injected with human cord blood infected with 2000 infectious units of the M81 EBV strain. Teriflunomide-treated animals received 20 mg/kg teriflunomide 3 times per week, starting on day 4. (**A**) Animals were euthanized after 28 days (first experiment; *N* = 4, each group) or 35 days (second experiment; *N* = 7, each group). Visible lesions were collected and analyzed histochemically. Lymphoma incidences for the two experiments were averaged. The significance of the difference in incidence between treated and untreated animals was calculated for each experiment; *p* values were then combined. (**B**–**E**) Histological analyses of a representative lymphoma from an untreated animal and the most advanced lesion found in a treated animal (both 28 days after infection). (B) Staining with hematoxylin and eosin (H&E) reveals that the lymphoma has invaded pancreatic tissue in the untreated animal. Images were taken with 2× or 10× objectives (20× and 100× magnification, respectively). (C) Antibodies to the B-cell marker CD20 and the T-cell marker CD3 reveal B cells and T cells in both the lymphoma and the lymphoid aggregate. (D) In situ hybridization for the EBV EBER RNAs and antibody staining for the EBV latency marker, EBNA2, reveal the presence of EBV-infected cells in both lesions. Immunohistochemical analysis of the spleen in (E) reveals the presence of T cells in both untreated and treated animals.

In two independent experiments, mice treated with teriflunomide failed to develop lymphomas (Figure 7A). In the first experiment, teriflunomide treatment (20 mg/ kg ip, three times a week, starting at day 4 after injection of EBV-infected cord blood cells) prevented the development of EBV-induced lesions of any kind in the cord-blood humanized mouse model. Histological and immunohistochemical analysis of the lesions that developed in teriflunomide-treated animals in the second experiment revealed only non-invasive lymphoid aggregates (Figure 7B). The top panels of Figure 7B show a typical large lymphoma (invading the pancreas) from an untreated control animal; the bottom panels show the most advanced lesion we detected (an EBV-infected non-invasive lymphoid aggregate) among the teriflunomide-treated animals. As is typical of the EBV-induced large lymphomas that have overwhelmed the T cell immune response in this humanized mouse model, the untreated tumor was highly invasive and consisted almost exclusively of B cells (which express the CD20 antigen), and a small minority of T cells (which express the CD3 antigen; Figure 7C). Significantly, teriflunomide did not prevent T cells from interacting with the small cluster of EBV-infected B cells (Figure 7C). The B cells in both the teriflunomide-treated and untreated lesions were largely infected with EBV, as revealed by the presence of EBV-encoded small RNAs (EBERs) and expression of EBNA2 (Figure 7D). Furthermore, the dose of teriflunomide used (which resulted in a blood level of 40 μg/ml) did not prevent engraftment of human T cells into the spleen of mice in this model (Figure 7E). These results indicate that teriflunomide treatment prevents EBV-infected B cells from forming invasive lymphomas in a humanized mouse model, where functional T cells have been shown to be required for control of EBV-induced lymphomas in the absence of teriflunomide treatment [42]. Thus, the T cell immunosuppressive effect of teriflunomide is counter-acted by its growth inhibitory effect on EBV-infected B cells in this model.

## DISCUSSION

Iatrogenic immunosuppression of transplant patients confers a greatly increased risk for EBV-induced LPD, which can be fatal. Development of LPD generally correlates with the overall degree of immunosuppression [43]. While reduction of immunosuppression can reverse EBV-induced LPD in many cases, it is not always feasible to reverse immunosuppression in patients who are also at high risk for organ rejection. Here we have investigated whether the immunosuppressive drug teriflunomide, a metabolite of leflunomide, prevents proliferation of EBV-transformed B cells *in vitro* and inhibits EBV-induced LPD in two different mouse models *in vivo*. We find that teriflunomide is surprisingly effective for inhibiting the growth of EBV-transformed B cells *in vitro* and treating EBV-induced lymphomas *in vivo*. In addition, we demonstrate that this drug prevents lytic EBV DNA replication in B cells both by blocking the ability of certain lytic inducing agents to activate expression of early lytic viral proteins, and by inhibiting lytic viral DNA replication. These results suggest that leflunomide/teriflunomide treatment of transplant recipients that have high EBV loads might reduce their risk of developing EBV-LPD. To our knowledge, this is the first demonstration that an immunosuppressive drug in clinical use can suppress EBV-LPD *in vivo* [43].

The inhibitory effect of teriflunomide on the proliferation and survival of EBV-infected LCLs *in vitro* appears to be multifactorial. At low doses, the effect is partially reversible by uridine supplementation and thus likely is due to the loss of activity of the major target of these drugs (cellular DHODH), and the resultant decrease in *de novo* pyrimidine synthesis. Previous studies have shown that rapid proliferation of activated lymphocytes in response to antigen stimulation requires *de novo* pyrimidine synthesis [17], and EBV-infected B cells hijack many of the same signaling pathways used by antigen-stimulated germinal center B cells to ensure their proliferation and survival [44]. In addition, consistent with a previous report showing that teriflunomide induces p53 activation [30], we found that low dose teriflunomide treatment of EBV-transformed LCLs increases p53 expression and apoptosis through an “on-target” mechanism.

Somewhat unexpectedly, we also discovered that teriflunomide greatly increases the amount of LMP1 expression in LCLs treated for one week. Importantly, although low level LMP1 expression is essential for proliferation and survival of established LCLs, higher level LMP1 expression, as found in teriflunomide treated LCLs, halts cell growth [45]. In addition, LMP1 over-expression *in vivo* may have an even more detrimental effect on LPD growth, as LMP1 increases T-cell mediated killing of EBV-infected B cells by enhancing MHC class I expression [46]. LMP1 expression is intricately regulated by the virus in LCLs through multiple different mechanisms. We found that teriflunomide increases both LMP1 RNA and protein levels. EBNA2, which we found at higher levels in teriflunomide-treated cells, as well as NFkB, which we also found to be elevated, are among the viral and cellular transcription factors that activate LMP1 transcription [47–49]. LMP1 is also regulated post-transcriptionally at the level of translation and autophagy-mediated degradation to ensure that levels of LMP1 are adequate to promote survival but not so high as to stop cell growth [29, 50, 51]. Although the exact mechanisms by which teriflunomide regulates LMP1 expression are not yet unraveled, the observed increase in LMP1 reflects an on-target effect (since it is reversible by uridine supplementation) and occurs at low doses of drug.

At higher doses *in vitro* (still clinically achievable in patients), we found that teriflunomide treatment produces an even more profound decrease in proliferation of EBV-transformed B cells, which is no longer reversed by uridine treatment. Thus these drugs also inhibit the proliferation of EBV-transformed B cells through “off-target” mechanisms. Although the precise mechanism(s) for these off-target effects on EBV-transformed B cells remain to be determined, they likely reflect the previously described ability of higher dose leflunomide/teriflunomide treatment to inhibit multiple different cellular tyrosine kinases, as well as AKT, S6 Kinase, NF-KB and STAT3 signaling [16, 23, 52]. Interestingly, however, we found increased, rather than decreased, NF-kappa B signaling in teriflunomide treated LCLs, presumably due to the increased expression of LMP1.

In addition, we show here for the first time that teriflunomide treatment blocks lytic EBV DNA replication in response to a variety of different lytic-inducing stimuli. While leflunomide has been previously reported to inhibit HCMV replication *in vitro*, the mechanism(s) by which it does so are not clear, and apparently occur downstream of intracellular viral DNA replication [22, 24]. Leflunomide/teriflunomide treatment also inhibits BK virus replication in newly infected cells; in this case, the drugs inhibit expression of the viral T antigen protein, which is required for viral replication [53]. A recent high-throughput screen for inhibitors of influenza virus replication identified a *de novo* pyrimidine synthesis inhibitor, A3, with effects on replication that were reversible by uridine [54]. A3 also inhibited the replication of a number of other RNA and DNA viruses [54]. These results suggest that leflunomide/teriflunomide might have broad anti-viral activity by inhibiting viral replication through its effects on *de novo* pyrimidine synthesis.

Interestingly, in the case of EBV, we found that teriflunomide can inhibit lytic EBV DNA replication through more than one mechanism, and through on-target and off-target effects. Teriflunomide blocks the ability of B-cell receptor stimulation, as well as TPA/sodium butyrate treatment, to induce even the very earliest step of lytic EBV reactivation (expression of the viral immediate-early lytic protein, BZLF1). While this effect occurs at low doses, it is apparently due to an “off-target” effect since it cannot be reversed by uridine.

In contrast, teriflunomide treatment does not prevent the ability of TGFβ to activate expression of the EBV immediate-early lytic protein, BZLF1, or the early lytic EBV protein, BMRF1, but prevents its ability to activate expression of a late structural protein (viral capsid antigen p18). Furthermore, consistent with the known inability of herpesvirus genes of the “late” class to be transcribed in the absence of lytic viral DNA replication [37], we found that the ability of TGF beta treatment to induce intracellular EBV DNA replication is prevented by teriflunomide. This latter effect appears to be at least partially due to an on-target effect of the drug, since late viral protein expression is partially rescued by uridine.

Perhaps the most striking finding in this study is our demonstration that clinically relevant doses of teriflunomide are quite effective for inhibiting the growth of EBV-transformed B cells in two different mouse models for EBV-LPD. In a cord-blood humanized mouse model where EBV-infected B cells and human T cells are co-engrafted, teriflunomide treatment (starting 4 days after injection of cells) did not prevent the engraftment of EBV-infected B cells or human T cells, but did block the ability of the EBV-infected B cells to form large invasive lymphomas. This result is particularly notable, since we have previously shown that human T cells inhibit the growth of the EBV-infected B cells in this model. Thus, the ability of teriflunomide to promote EBV-induced lymphomas via its T cell immunosuppressive effect is clearly outweighed by its ability to inhibit the proliferation and/or survival of EBV-infected B cells.

In addition, in a xenograft model for EBV-LPD, even when teriflunomide therapy was delayed until 8 days after LCL injection into mice (at which point small tumors were already palpable, and could also be visualized by luciferin scanning; Figure 6), the drug still greatly inhibited the growth of the LCL-induced lymphomas. In this xenograft model, only the ability of teriflunomide to inhibit proliferation of latently-infected EBV-positive B cells contributes to its anti-tumor effect, since EBV cannot infect mouse cells, and all injected LCLs at the start of the experiment were already EBV-infected.

In summary, our investigations here, using both *in vitro* as well as *in vivo* systems to model the effects of the FDA-approved leflunomide metabolite teriflunomide on latent and lytic EBV B-cell infection, suggest that these drugs may be surprisingly effective for treating both latent and lytic EBV infection in humans. The most obvious potential clinical use of leflunomide/teriflunomide for control of EBV infection in humans would be in the transplant recipient population, since such patients require immunosuppression in any event, and are at high risk for developing EBV-driven LPD. Nevertheless, these drugs might also be useful in rare cases of fulminant infectious mononucleosis, in which the clinical symptoms are due not only to uncontrolled proliferation of virally-infected B cells, but excessive T cell activation in response to the EBV-infected B cells. In such cases, short term leflunomide/teriflunomide therapy might not only reduce clinical symptoms by inhibiting T cell proliferation, but could also prevent the expansion of EBV-infected B cells.

A strength of our work is its demonstration that teriflunomide inhibits spontaneous lymphomagenesis in a human cord blood model of LPD. Limitations of our studies include the examination of only one cell line in xenografts, and the relatively short duration of the teriflunomide treatment in both mouse models. Long-term studies of the effect of leflunomide treatment on LCL growth in our animal models would help determine whether lymphomas become resistant to treatment. Finally, clinical trials will be required to determine if leflunomide/teriflunomide treatment is safe and effective, alone or in combination with other immunosuppressive drugs, before recommending such therapy for selected EBV-associated diseases in humans.

## MATERIALS AND METHODS

### Cell lines and culture

Mutu I (a gift from Alan Rickinson) and Kem I (a gift from Jeffrey Sample) are EBV-positive Burkitt lymphoma cell lines and were cultured in RPMI (GIBCO) supplemented with 10% FBS and 1% penicillin-streptomycin. D4 LCL is an EBV-transformed (B95.8) B cell lymphoblastoid cell line (LCL) derived from the peripheral blood leukocytes of anonymous donors, obtained from the American Red Cross [55]. M81-Luc LCLs were obtained by infecting CD34-depleted human cord blood (AllCells) with M81-Luc virus, described below, and selecting immortalized cells. HONE-Akata (a gift from Lawrence Young, University of Birmingham) is an EBV-superinfected (Akata strain) epithelial cell carcinoma cell line. These cells were grown in DMEM (GIBCO) supplemented with 10% FBS (Hyclone) and 1% penicillin-streptomycin (GIBCO).

### Construction of an M81 recombinant virus that constitutively expresses the luciferase gene

We constructed a recombinant M81 strain with constitutive luciferase activity (B1129) by introducing a luciferase gene (pGL4.5, Promega) into the M81 BAC [56] via homologous recombination [57] at the SmaI restriction site of the BXLF1 coding region (coordinate 131,127 on the M81 genome, GenBank accession number KF373730.1). The expression of the luciferase gene on this virus is driven by a CMV early enhancer/chicken beta actin (CAG) promoter. The disruption of BXLF1 gene has previously been confirmed not to interfere with the growth of LCLs [58]. Infectious virions were harvested from 293 cells (ATCC) stably infected with M81-Luc virus.

### *In vitro* drug treatment studies

### Growth assays

D4 LCLs were plated at 2 × 10^5^ cells/ml on day 0 and harvested on day 7. One hour after plating, cells were treated with dimethyl sulfoxide control (DMSO; Sigma-Aldrich); or with 2.5 to 70 μg/ml teriflunomide (A771726; CalBiochem in Figure 1A; ENZO Life Sciences in all others) dissolved in DMSO. The final DMSO concentration in control and treatment groups was 0.1%. Uridine-treated cells were given 150 μM uridine one hour prior to teriflunomide or DMSO treatment. Growing cells were expanded into fresh medium containing DMSO or teriflunomide, with or without 150 μM uridine (Sigma-Aldrich), as needed. Cells treated with teriflunomide in Figure 1A were given fresh drug every 24 hours. Cells were counted using trypan blue exclusion (Figure 1A) or relative cell titers were determined using Cell Titer Glo as instructed by the manufacturer (Promega; Figure 1B).

### Drug response studies

D4 LCLs were treated for 7 days with 6.5 to 70 μg/ml of teriflunomide, added one hour after cells were plated with or without uridine at 3 × 10^5^ cells/ml. Growing D4 LCLs were expanded into fresh medium supplemented with drugs as needed. All D4 LCLs were given fresh teriflunomide on day 3, day 5 (Figure 3C only), and day 6.5. M81-Luc LCLs, Mutu I, and Kem I cells at 2–5 × 10^5^ cells/ml were treated for 3 days with 6.5 to 50 μg/ml teriflunomide, 150 μM uridine, or 0.1% DMSO control, on day 0 only, and were not expanded. The following reagents were added one hour after teriflunomide treatment to induce lytic EBV reactivation: phorbol 12-myristate 13-acetate (TPA; 20 ng/ml; Sigma-Aldrich), sodium butyrate (3 mM; Sigma-Aldrich), anti-human IgG (10 μg/ml; Sigma-Aldrich), anti-human IgM (6.8 μg/ml; Southern Biotech), or TGFβ (5 ng/ml; R&D Systems). HONE-Akata cells were treated with TPA and sodium butyrate as above on day 0 and were collected on day 3.

### Cell death assay

Cells were plated at 3 × 10^5^ cells/ml on day 0 and treated with teriflunomide or control DMSO. DMSO-treated wells required expansion on day 2. All cells received fresh drug/DMSO and medium on day 3. On day 5, cells were collected, washed twice with PBS, resuspended in fresh medium without drugs or DMSO, and plated at 3 × 10^5^ cells/ml. Caspase 3/7 activity was measured on day 6 using 25 μl sample and 25 μl Caspase Glo 3/7 (Promega).

### Immunoblot analysis

Cell lysates were harvested in Sumo lysis buffer including protease inhibitors (Roche) as described previously [59], except cells were washed only once with PBS (DPBS, GIBCO) prior to resuspension. Protein concentration was determined using the Sumo protein assay (Biorad), and proteins were separated in SDS-10% polyacrylamide gels and then transferred onto a nitro-cellulose membrane (0.22 μm, used only for p18, Maine manufacturing; or 0.45 μm, GVS North America). Membranes were blocked in PBS containing 5% non-fat dry milk (Roundy’s), and 0.1% Tween 20 (Sigma-Aldrich). Membranes were then incubated in PBS/5% Bovine Serum Albumin (Research Products International; for Exalpha antibodies), PBS/5%BSA/0.1%Tween 20 (Cell Signaling antibodies) or PBS/5% milk/0.1% Tween 20 (all other antibodies). The following primary anti-human antibodies were used: anti-EBNA2 (mouse, #90543, clone PE2, Abcam, 1:500); anti-LMP1 (mouse, #78113, clones CS1-4, Abcam, 1:500); anti-EBNA3A (sheep polyclonal, Abcam, 1:2000); anti-EBNA3c (sheep polyclonal, Exalpha Pharmaceuticals, 1:2000); anti-β-actin (mouse, A5441, clone AC-15, Sigma, 1:5,000); anti-NFkB2p100/p52 (rabbit, #3017, Cell Signaling, 1:1000); anti-phosphoNFkBp65 (ser536, rabbit, #3303, clone 93H1, Cell Signaling 1:1000); anti-cMYC (rabbit, #32072, clone Y69, Abcam, 1:10,000); anti-Cyclin E (mouse, #sc-247, clone HE12, Santa Cruz, 1:250); anti-PARP (rabbit, #9542, Cell Signaling, 1:1000); anti-p53 (mouse, clone DO-1, Santa Cruz, 1:1000); anti-BZLF1 (mouse, sc-53904, BZ1, Santa Cruz, 1:500); anti-BMRF1 (mouse, MAB8186, clone R3, Millipore, 1:3,000); anti- EBV p18 protein (goat polyclonal; Thermo Fisher Scientific, 1:2000); and anti-glyceraldehyde-3-phosphate dehydrogenase (GAPDH)-horseradish peroxidase (HRP) antibody (A00192; Genscript; 1:4,000). The secondary antibodies used were horseradish peroxidase (HRP)-labeled goat anti-mouse antibody (Thermo Fisher Scientific, 1:5,000), goat anti-rabbit antibody (Thermo Fisher scientific, 1:5,000); rabbit anti-sheep antibody (Santa Cruz, 1:5000); and donkey anti-goat antibody (Santa Cruz, 1:5000). Blots were developed with the Pierce ECL Western Blotting Kit (Thermo Fisher Scientific).

### Reverse transcriptase quantitative PCR (RT-qPCR) analysis

Total RNA was extracted using the RNA-Bee reagent (Tel-Test Inc., catalog # Cs-104B) from D4 LCLs treated with 20 μg/ml teriflunomide or DMSO control for 7 days. The extracted RNA was then DNAse treated, followed by reverse transcription using random primers and GoScript Reverse Transcriptase (Promega, catalog # A5000). Real-time PCR was performed on the reverse transcribed cDNA using the iTaq Universal SYBR Green mix (Bio-Rad, catalog # 1725121) in Biorad CFX96 machine.1.5 μl of cDNA was used for 40 cycles (15 seconds at 95°C and 30 seconds at 60°C), using primers that will detect LMP1 transcript originating from both TR and ED-L1 promoters (LMP1-TR + EDL1, forward primer: 5′- TGAGTAGGAGGGTGA – 3′ and reverse primer: 5′- CTATTCCTTTGCTCTCATGC - 3′) and beta-Actin transcript (forward primer: 5′ – GCC GGGACCTGACTGACTAC- 3′ and reverse primer: 5′ - TTC TCCTTAATGTCACGCACGAT– 3′). Beta-actin was used as a housekeeping gene, and transcripts were quantified using the delta-delta Cq methods for each time point.

### Viral DNA replication quantitative PCR assay

Intracellular DNA from 10^6^ treated Mutu I cells (treatments as indicated in the Figure legend) was harvested, purified, and quantified as described [60].

### Mouse studies

All animal experiments were approved by the University of Wisconsin-Madison Institutional Animal Care and Use Committee (IACUC) and conducted in accordance with the NIH Guide for the Care and Use of Laboratory Animals [61]. Immunodeficient NSG (NOD/LtSz-*scid/IL2R*g^null^) mice were bred at UW-Madison from stocks purchased from The Jackson Laboratory.

### Xenograft studies

NSG mice were injected subcutaneously in the flanks with 5 × 10^6^ M81-Luciferase (M81-Luc) lymphoblastoid cells. Mice were treated with 20 mg/kg teriflunomide intraperitoneally three times a week, beginning on day 8 and sacrificed on day 21 post-infection (a total of six injections). To determine tumor size, mice were injected intraperitoneally with the luciferase substrate luciferin (150 mg/kg; Gold Biotechnology) and scanned using an IVIS Spectrum *in vivo* imaging system. The light produced by the luciferase was quantified using Living Image software (PerkinElmer).

### Production of infectious virus

Infectious viral particles were produced from 293 cell lines stably infected with the M81 virus [56] or the M81-Luc virus following transfection with EBV BZLF1 and GP110 expression vectors as previously described [62]. EBV was titered on Raji cells (ATCC) using the Green Raji cell assay as previously described [62].

### Humanized mouse model

Commercially purchased CD34-depleted human cord blood mononuclear cells (AllCells, LLC., CB117) were infected with M81 strain virus using 2000 infectious units. Cord blood was initially exposed to the virus *in vitro* for 1.5 hours and then 10 to 25 million cells were injected intraperitoneally (i.p.) into 3–5 week old NSG mice. Mice were treated with 20 mg/kg teriflunomide starting on day 4, three times a week. Mice were euthanized on day 35 (Experiment 1) or 28 (Experiment 2). Tumor size was quantitated by dissecting and weighing grossly visible tumor tissue.

### Tissue Analysis

Following euthanasia of EBV-infected humanized mice, multiple different organs (including the lungs, spleen, pancreas, liver, gall bladder, and mesenteric fat) were formalin fixed. Paraffin-embedded sections were then analyzed using a variety of techniques to determine if animals had persistent EBV infection and/or EBV-positive lymphomas, and to assess the viral protein expression pattern. Sections were stained with hematoxylin (Shandon Instant Hematoxylin, Shandon Lipshaw) and eosin (Eosin Y, Sigma-Aldrich), hybridized *in situ* with probes for EBV EBER RNAs (PNA ISH Detection Kit; DakoCytomation), or analyzed immunohistochemically using the following anti-human antibodies: anti-CD20 (mouse, clone H1(FB1), BD Pharmingen), anti-CD3 (mouse, clone F7.2.3; Dako), and anti-EBNA2 (mouse, Abcam) as previously described [62]. Images were taken with 2× and 10× objectives (20× and 100× magnification, respectively) using an Olympus BX53 microscope.

### Statistics

Mstat Software (http://mcardle.wisc.edu/mstat/download/index.html) was used to statistically analyze the data. For tumor formation comparison, the *p* value was calculated using a two-tailed Fisher exact test.
